# Noncanonical PD-1/PD-L1 Axis in Relation to the Efficacy of Anti-PD Therapy

**DOI:** 10.3389/fimmu.2022.910704

**Published:** 2022-05-18

**Authors:** Yiru Long, Xiaolu Yu, Runqiu Chen, Yongliang Tong, Likun Gong

**Affiliations:** ^1^ State Key Laboratory of Drug Research, Shanghai Institute of Materia Medica, Chinese Academy of Sciences, Shanghai, China; ^2^ University of Chinese Academy of Sciences, Beijing, China; ^3^ Department of Pharmaceutics, Wuya College of Innovation, Shenyang Pharmaceutical University, Shenyang, China; ^4^ Zhongshan Institute for Drug Discovery, Shanghai Institute of Materia Medica, Chinese Academy of Sciences, Zhongshan, China

**Keywords:** noncanonical PD-1/PD-L1 axis, T-cells, tumor cells, myeloid cells, anti-PD therapy

## Abstract

With programmed death 1/ligand 1 (PD-1/PD-L1) as the cornerstone, anti-PD antibodies have pioneered revolutionary immunotherapies for malignancies. But most patients struggled to respond to anti-PD owing to primary or acquired resistance or even hyperprogression, pointing to more efforts needed to explore this axis. PD-1 constrains T-cell immunoreactivity *via* engaging with PD-L1 of tumor/myeloid cells is the canonical PD-1/PD-L1 axis function mode. Studies are increasingly aware of the impact of noncanonical PD-1/PD-L1 expression in various cancers. PD-L1 induced on activated T-cells ligates to PD-1 to mediate self-tolerance or acts on intratumoral myeloid cells and other T-cells, affecting their survival, differentiation and immunophenotyping, leading to tumor immunosuppression. Myeloid PD-1 interferes with their proliferation, differentiation, cytokine secretion and phagocytosis, mediating remarkable pro-tumor effects. Tumor cell intrinsic PD-1 signaling has diverse functions in different tumors, resulting in pro-proliferation or proliferation inhibition. These nonclassical PD-1/PD-L1 functions may be novel anti-PD mechanisms or causes of treatment resistance. This review highlights the nonnegligible role of T-cell-intrinsic PD-L1 and tumor/myeloid PD-1 in the cell interplay network and the complex impact on the efficacy of anti-PD antibodies. Reconsidering and rational utilization of the comprehensive PD-1/PD-L1 axis could cumulate breakthroughs in precision treatment and combination for anti-PD therapies.

## Introduction

Programmed death-1 (PD-1) was discovered in 1992 as an apoptosis-associated gene (1). Subsequent studies identified PD-1 as a negative regulatory immune molecule to maintain self-tolerance, containing cytoplasmic immunoreceptor tyrosine-based inhibitory motif (ITIM) and switch motif (ITSM), and predominantly expressed in T/B-lymphocytes (2–5). Programmed death ligand 1 (PD-L1) was discovered in 1999 as a novel member of the B7 family (6). In 2000, PD-L1 was found to be a ligand for PD-1, which inhibits T-cell proliferation (7). Later studies revealed that PD-L1, abundantly expressed on tumor and myeloid cells, induces T-cell apoptosis and immunosuppression to achieve tumor escape, and is a potential tumor therapeutic target (8–10).

Since 2012, numerous clinical reports (11–19) have shown the unprecedented efficacy of anti-PD antibodies for the treatment of metastatic bladder cancer, renal-cell cancer, colorectal cancer (CRC), non–small-cell lung cancer (NCSLC), and melanoma, etc. Frustratingly, durable responses to PD-1/PD-L1 antibodies are only achieved in about 10-40% of patients, with the majority not benefiting (20). In parallel to the necessity to address resistance and hyperprogression, immune-related adverse events cannot be neglected (21, 22), emphasizing in-depth investigation of the physiological-pathological functions and regulatory mechanisms of the PD-1/PD-L1 axis is paramount.

This review focuses on significant advances in the nonclassical PD-1/PD-L1 axis, summarizes and discusses the roles of T-cell-intrinsic PD-L1 and myeloid/tumor cell-intrinsic PD-1 in cancer progression and the complex implications for anti-PD efficacy, hoping to inspire more rational anti-PD drug design and combination strategies.

## Classical Expression and Location of PD-1/PD-L1

Generally, the PD-1/PD-L1 axis is involved in tumor immune escape *via* antigen-presenting cell (APC) or tumor cell surface PD-L1 mediating suppression of PD-1^+^ CD8^+^ T-cells and blocking PD-1/PD-L1 ligation can reinvigorate anti-tumor adaptive immunity (20, 23). The classical PD-1/PD-L1 axis is the main attraction for drug development.

### PD-1 on T-Cells

Earlier studies concluded that PD-1 was mainly expressed on thymic and splenic T-cells (3, 24, 25) and that PD-1 ligation on CD8^+^ T-cells inhibits naive-to-effector differentiation, cytotoxicity, proliferation, and survival during chronic infections and tumor progression (26–32). PD-1/PD-L1 blockade rescues CD8^+^ T-cells from exhaustion or dysfunction (26, 29, 32). Increased PD-1^+^ CD8^+^ T-cells are positively correlated with anti-PD responses (33, 34). Notably, PD-1 expression does not necessarily determine T-cell exhaustion (35). Partial PD-1 intermediate T-cells maintain proliferation and interferon-γ (IFN-γ)/tumor necrosis factor-α (TNF-α) secretion and show well potential for anti-PD reinvigoration (36, 37). PD-1 signaling also affects other T-cell subsets. PD-L1 engagement on CD4^+^ T-cells affects cytokine secretion and induces differentiation into regulatory T-cells (Tregs) (38–40). Follicular helper T-cells, natural killer T-cells (NKT) and γδ T-cells also reduce antitumor activity or exert regulatory functions due to PD-1 function (41–45). Recent advances demonstrated that PD-1 ligation also regulates the metabolic reprogramming and migration of T-cells (45–47).

### PD-L1 on Tumor Cells and Myeloid Cells

PD-L1, abundantly expressed on tumor or myeloid cells, engages on antitumor T-cells to accelerate their apoptosis and malfunction (8, 10). Anti-PD-L1 antibodies are effective in reversing the tumor immunosuppression microenvironment (9, 10). The contribution of host and tumor PD-L1 to the efficacy of anti-PD blockade remains controversial. Recently, researchers suggested that PD-L1 of host myeloid cells mainly determines the efficacy of PD-L1 antibodies (48–51). Tumor cell-derived PD-L1 exosomes were also shown to inhibit the anticancer activity of T-lymphocytes (52). Additionally, complicated membranal protein interactions and intracellular signaling of PD-L1 were revealed, suggesting there are multiple unresolved gaps in PD-L1 function. The cis-CD80/PD-L1 interactions on APCs impede PD-L1/PD-1 and CD80/cytotoxic T-lymphocyte-associated antigen-4 (CTLA-4) inhibitory signaling but do not affect CD28 co-stimulatory signaling (53, 54). Ongoing studies have indicated that PD-L1 as a receptor could transmit signals and impact the anti-apoptosis, chemotaxis, neoantigen presentation, and glycolysis of tumor cells or APCs (55–59). Moreover, cytoplasmic or nuclear translocation of PD-L1 could modulate genomic stability, DNA damage response, pyroptosis, and gene transcription of tumor cells (60–63).

## PD-L1 Expressed on T Cells

Although the T-cells-intrinsic-PD-L1 has been insufficiently studied, in fact, as early as when PD-L1 was identified, Lieping Chen’s team already found that although PD-L1 was not expressed in freshly isolated human and mouse T-cells, but could be upregulated in activated T-cells (6, 64), especially in CD4^+^ T-cells and CD45RO^+^ memory T-cells (65). They also found that autoantibodies against PD-L1 in rheumatoid arthritis patients acted on primary CD4^+^ T-cells to promote apoptosis of activated CD4^+^ T-cells in an interleukin-10 (IL-10)-dependent manner. Subsequently, *via* PD-L1-deficient mice, researchers found that PD-L1 depleting led to increased CD4^+^ T-cell cytokine production, increased CD8^+^ T-cell expansion and cytotoxicity, and increased intrahepatic accumulation and survival of CD8^+^ T-cells, as well as impaired autoimmune tolerance (25, 66, 67). Su-Kil Seo et al. reported that T-cell-associated PD-L1 interacted with PD-1 on T-cells *via* the alloreactive T–T interaction, resulting in reduced T-cell proliferation and IFN-γ and IL-2 production (68). And Nuriban Valero-Pacheco et al. observed that PD-L1^+^ CD8^+^ T-cells were related to a lower T-cell proportion in patients infected with the H1N1 virus (69). These early findings revealed that PD-L1 was expressed on activated T cells and may also act as a receptor to receive signals that affect T-cell activation and self-tolerance maintenance.

Nonetheless, several researches conducted during the same period discussed that T-cell-intrinsic-PD-L1 functions appeared to contradict the preceding conclusions. Oezcan Talay et al. demonstrated that activation and proliferation of PD-L1^-/-^ CD8^+^ T-cells in the initial phase against influenza virus was impaired and that PD-L1 expressed on naive T-cells was required for T-cell-mediated dendritic cell (DC) maturation (70). Seung-Joo Lee and colleagues also found a significant increase in PD-L1 on CD4^+^ T-cells during *Salmonella* infection and that PD-L1-deficiency did not affect specific antibody production and CD4^+^ T-cell expansion but affected CD4^+^ T-cell maturation and function (71). For anti-tumor immunity, Vesna Pulko et al. found that PD-L1 upregulation on primed T-cells helped effector T-cells survive the contraction phase, but anti-PD-L1 hindered T-cell survival (72). Their results also showed that PD-L1-deficient CD8^+^ T-cells were more sensitive to cytotoxicity, whereas adoptive PD-L1-deficient T-cell therapy was ineffective in restraining the growth of B16-OVA tumors. According to Asim Saha et al., upregulated PD-L1 expression in donor T-cells promoted graft-versus-host responses (73). PD-L1-deficient T-cells had fewer gut homing receptors, produced fewer inflammatory cytokines, enhanced apoptosis and multiple bioenergetic pathways.

Additional studies have reported puzzling roles for anti-PD-L1 antibodies in antitumor, anti-infection and anti-autoimmune diseases (74–77). For example, two publications found that *Listeria* infection enhanced T-cell PD-L1 expression, whereas PD-L1 antibody blockade selectively obstructed the anti-intracellular bacterial responses of CD8^+^ T-cells (75, 76). Notably, partial anti-PD-L1 antibodies caused apoptosis of PD-L1^+^ T-cells, even PD-1-knockout T-cells, by activating p38 MAPK, and that such antibodies failed to suppress B16-OVA and RENCA tumor growth *in vivo* (77). And these PD-L1^+^ T-cells inhibited the apoptosis of activated CD8^+^ T-cells *via* altering phosphorylation of p38 MAPK through intracellular interactions with DNA-PK. These results have sparked a debate about whether T-cell-intrinsic PD-L1 regulates the immune system positively or negatively. Researchers are reminded to focus on the complexity of T-cell-intrinsic PD-L1 function, where the different phases of immune responses, immune cell crosstalks, unexpected protein interactions, and specific anti-PD-L1 functions all require careful exploration.

**Figure 1:**
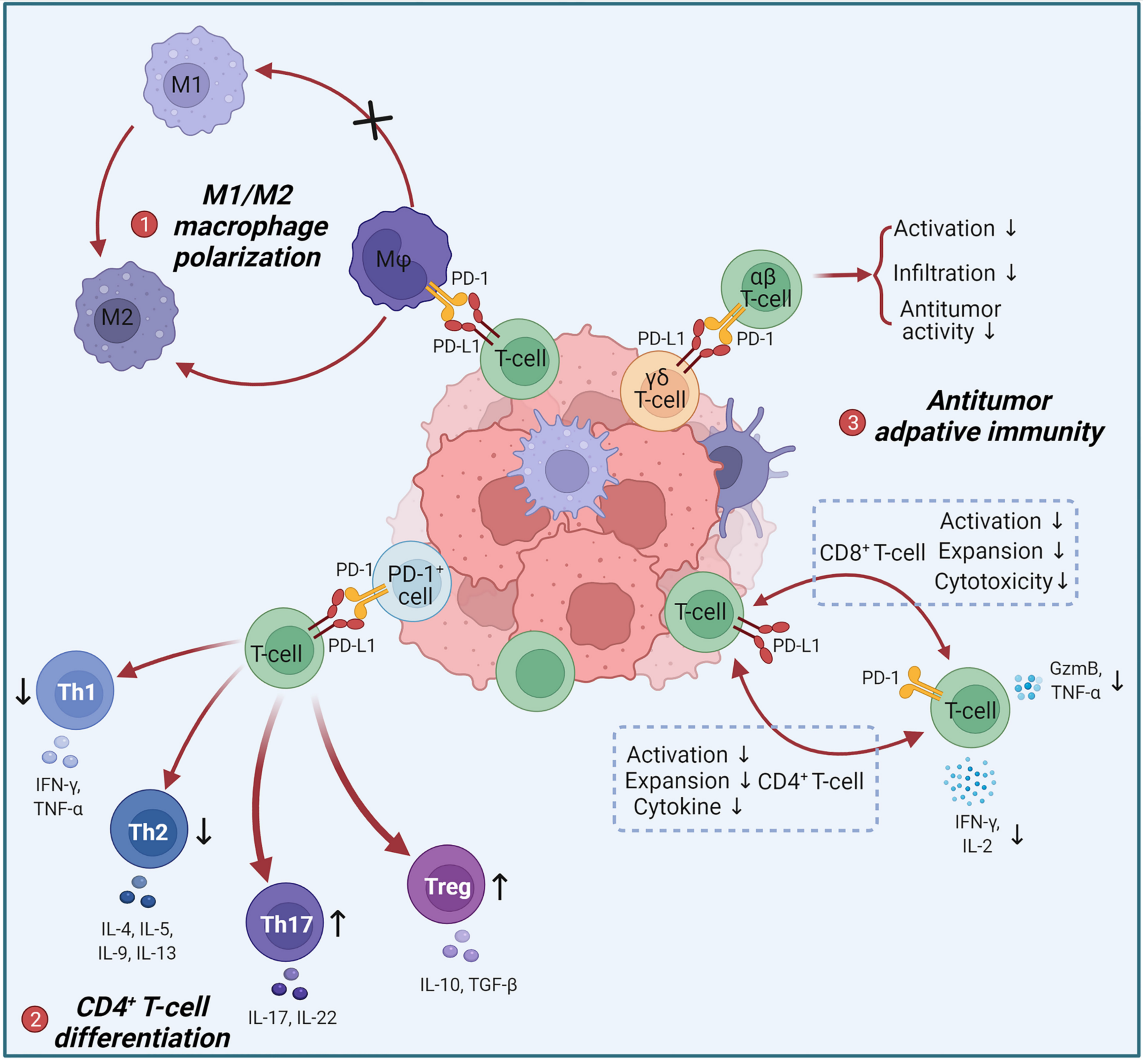
Negative regulation of anti-tumor immune responses by T-cell intrinsic PD-L1. (1) Tumor-infiltrating PD-L1^+^ T-cells act on intratumoral PD-1^+^ macrophages to induce M2 polarization. M2 can promote tumor progression. (2) PD-1^+^ cells act on PD-L1^+^ T-cells to limit the differentiation of Th1 and Th2 and promote the differentiation of Th17 and Tregs, leading to an increase in pro-tumor immunosuppressive factors such as IL-10, IL-17 and TGF-β. (3) PD-L1^+^ T-cells and PD-1^+^ T-cells achieve bidirectional signaling through PD-1/PD-L1 interactions, resulting in bidirectional immunosuppression: inhibition of CD4^+^ T-cells activation, expansion and cytokine secretion; inhibition of CD8^+^ T-cells activation, expansion and cytotoxicity. PD-L1^+^ γδ T-cells suppress the activation, intratumoural infiltration and antitumor activity of αβ T-cells *via* the PD-L1/PD-1 axis.

In recent years, important progresses have been made regarding the expression pattern and immunomodulatory function of T-cell-intrinsic PD-L1 (**Figure 1**). Donnele Daley and colleagues found that PD-L1 expression switched positive in approximately 50% of γδ T-cells in human and murine pancreatic ductal adenocarcinoma (PDA), and that blocking PD-L1 in γδ T-cells enhanced activation and infiltration of CD4^+^ and CD8^+^ T-cells (78). Subsequently, Brian Diskin et al. used extensive experiments to elucidate the regulatory role and mechanisms of T-cell-intrinsic PD-L1 in PDA tumors (79). They detected that PD-L1 was expressed on >50% of intratumoral T-cells in the orthotopic PDA model and increased with progressive oncogenesis. And 63% of T-cells in B16 tumors and 17% of T-cells in MCA38 tumors also expressed PD-L1. Intriguingly, the highest PD-L1 expression in human PDA was found in T-cells, rather than in tumor cells or macrophages as commonly thought. Based on the fact that conditional ablation of PD-L1 in T-cells promoted adaptive anti-tumor responses and activated macrophages, they elucidated that PD-L1^+^ T-cells reinforce an immune tolerant environment to accelerate carcinogenesis through three ways: (1) PD-L1 engagement on T-cells inhibits Th1 differentiation but promotes Th17 differentiation *via* a STAT3-dependent manner, while inducing an anergic phenotype in CD8^+^ T-cells (2) PD-L1^+^ T-cells deliver inhibitory signals to PD-1^+^ T-cells; (3) PD-L1^+^ T-cells engage PD-1^+^ macrophages to promote M2-preference differentiation. Giorgia Fanelli et al. proved that PD-L1 ligation accompanied by CD3/TCR stimulation tended to transform memory T-cells but not naive T-cells into highly suppressive Tregs by triggering the PD-L1 intracellular pathway as reducing ERK phosphorylation and decreasing AKT/mTOR/S6 signaling (80). And Fabienne Mazerolles et al. suggested that T-cell proliferation was correlated with the PD-L1 expression of activated naive CD4^+^ effector T-cells regulated by DCs and Tregs (81). Thus, T-cells-intrinsic PD-L1 has bidirectional signaling that affects CD4^+^ T-cell and macrophage differentiation and attenuates cytotoxic T-cell effects to drive immune tolerance.

In addition, numerous evidences have shown peripheral or tumor-infiltrating PD-L1^+^ T-cell levels have the potential to be served as clinical indicators. Two papers reported that melanoma patients had greater PD-L1^+^ circulating T-cell levels than healthy volunteers, and PD-L1^+^ CD8^+^ T-cells were raised in disease relapsed or disease-related dead patients (82, 83). Furthermore, PD-L1^+^ circulating CD4^+^/CD8^+^ T-cells may be a predictive biomarker for anti-CTLA-4 therapy resistance. Bruktawit A. Goshu et al. demonstrated that anti-PD-L1 (Avelumab) targeting PD-L1^+^ HIVGag-specific-CD8^+^ T-cells combined with rhIL-15 enhanced CD8^+^ T-cell activity during HIV infection (84). Xia Li et al. identified dynamic fluctuations in PD-L1 on CD4^+^/CD8^+^ T-cells around the partial mission phase of type 1 diabetes and suggested PD-L1 may be a potential target for prolonging this phase (85). Several analyses (86–91) of patient samples involving ovarian cancer, NSCLC, and chronic lymphocytic leukemia (CLL) suggested an association between low circulating or infiltrating PD-L1^+^ CD8^+^ T-cells and prolonged survival, but high PD-L1^+^ CD8^+^ T-cell levels predicted a better anti-PD-1/PD-L1 therapy response. Among them, Libin Zhang et al. used a cohort of 378 NSCLC cases to speculate that CD8^+^ PD-L1^+^ TILs might indicate a hot but immunosuppressive tumor microenvironment with a high mutation burden (90). Nikolaos Ioannou et al. found that avadomide, *via* triggering IFN signaling in T-cells to increase PD-L1 expression on T cells, reprogramed patients’ T-cells, which complements PD-L1/PD-1 blockade (91).

## Tumor Cell-Intrinsic PD-1

Given the predominant biofunction of PD-1 on T-lymphocytes, T-cell-extrinsic PD-1 has been largely neglected. Yet persistent studies focusing on the non-classical PD-1 are shedding further light on previously incomprehensible biological and clinical phenomena. Currently, PD-1 has been identified to be expressed on various clinical tumor cells or tumor cell lines of CRC, melanoma, hepatocellular carcinoma (HCC), NSCLC and PDA (92–102). However, the ramifications of tumor cell-intrinsic PD-1 on oncogenesis have sparked much controversy.

**Figure 2:**
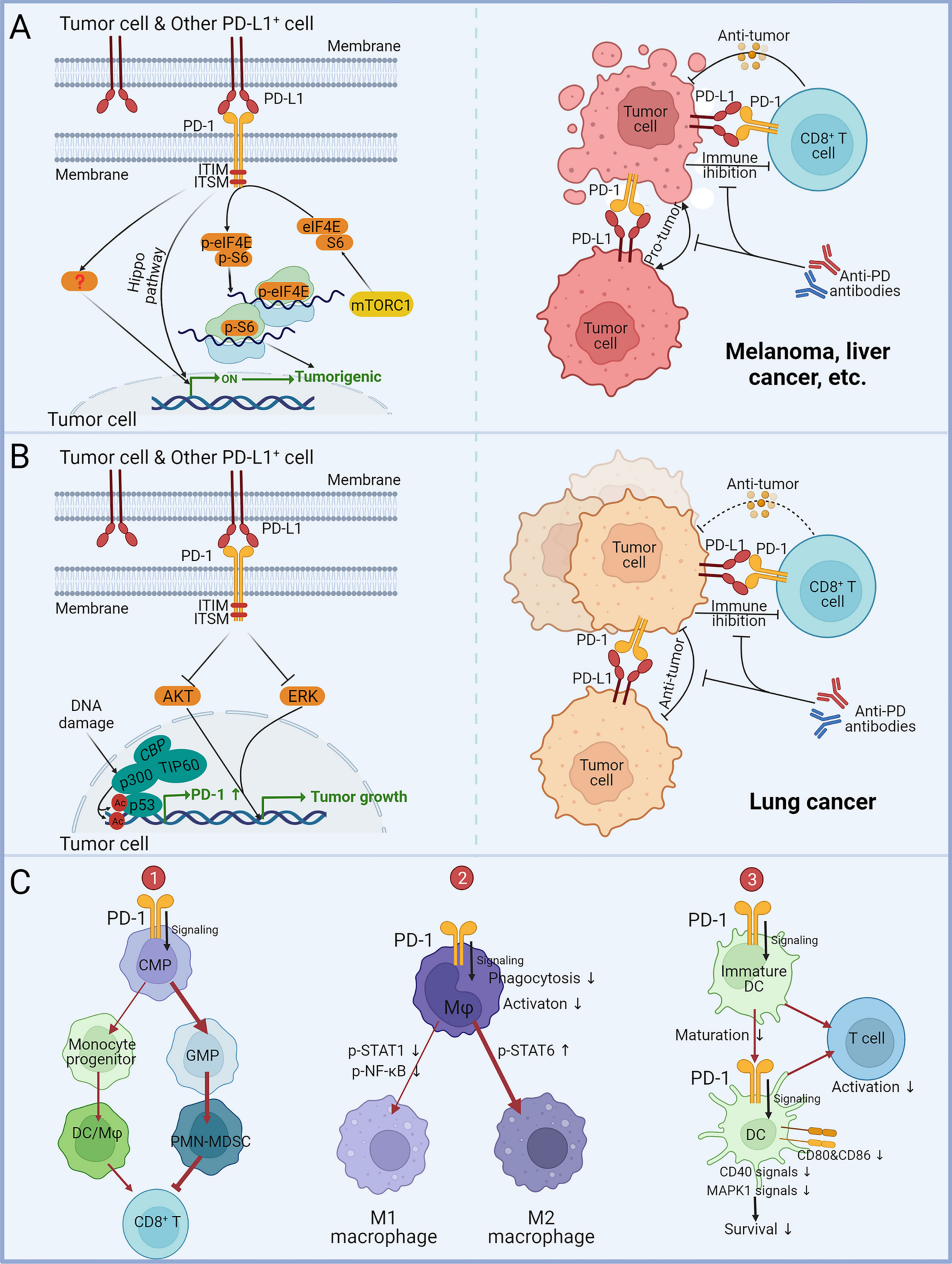
Impact of tumor and myeloid cell-intrinsic PD-1 on tumor progression. **(A)** The effect of tumor cell-intrinsic PD-1 on melanoma, liver cancer, and other malignancies. PD-L1 expressed by tumor cells or other cells acts on PD-1^+^ tumor cells to mediate PD-1 signaling in tumor cells *via* ITIM and ITSM. The Hippo pathway and phosphorylation of mTOR downstream effector molecules eIF4E and S6 can enhance tumor-promoting gene transcription and protein expression. Anti-PD antibodies can block the PD-1/PD L1-mediated tumor promotion independent of adaptive immunity. **(B)** The role of tumor cell-intrinsic PD-1 in lung cancer. PD-L1 expressed by tumor cells or other cells acts on PD-1^+^ tumor cells to suppress tumor growth by dampening AKT and ERK signaling. Acetylation of p53 promotes gene transcription of PD-1. Anti-PD antibodies block PD-1/PD-L1-mediated tumor suppression, leading to hyperprogression in immunocompromised patients. **(C)** Effects of myeloid PD-1 on cell development, differentiation and function. (1) PD-1 expression promotes common myeloid progenitors (CMP) differentiation into granulocyte/macrophage progenitors (GMP), leading to increased MDSCs in granulocyte lineages and suppressing the activity of anti-tumor CD8^+^ T-cells. (2) PD-1 suppresses M1 polarization by reducing STAT1 and NF-κB phosphorylation and promoted M2 polarization by increasing STAT6 phosphorylation. (3) PD-1 suppresses DC maturation, survival and co-stimulatory molecules expression, consequently downregulating antigen-specific T-cell activity.

For most oncological diseases, tumor cell-intrinsic PD-1 augmented cancer advancement independently of adaptive immunity (**Figure 2A**). Sonja Kleffel and colleagues earlier identified that preferential expression of PD-1 by ABCB5^+^-melanoma cells mediated increased tumorigenic capacity (94). Then they noticed that 3.5% to 16.5% of clinical melanoma cells expressed PD-1, and PD-1 positive frequencies ranged from 11.3% to 29.5% in eight human melanoma cell lines and from 6.6% to 9.4% in two murine melanoma cell lines (95). Through PD-1 knockdown/overexpressing B16 phenotype in NSG, they determined tumor PD-1 on B16 promoted tumorigenesis independently of immunity. By mutating the tyrosine sites of ITIM and ITSM, it was determined that melanoma-PD-1-driven tumorigenesis required the interactions between melanoma-PD-1 and host/melanoma-PD-L1 to initiate the PD-1 intracellular signaling *via* the mTOR pathway. Hui Li et al. later reported that five HCC cell lines and clinical HCC tissues contained subpopulations upregulating PD-1 (96). PD-1 interacted with and promoted phosphorylation of the mTOR effectors eIF4E and S6 to enhance tumor growth. Ning Pu et al. believed that PD-1 of PDA cells promoted tumor growth and apoptotic resistance *via* PD-L1 ligation and Hippo signaling (99).

These teams also showed that blocking PD-1 inhibited the growth of xenografts in immunodeficient mice, and innovative combination strategies have been proposed and practiced (95, 96, 99). Li Hui et al. tried mTOR inhibitors in combination with anti-PD-1 to accomplish more durable and synergistic tumor regression (96). Ning Pu et al. found that Hippo pathway inhibitors together with anti-PD-1 treatment showed remarkable tumor eradication (99). Besides, the two teams highlighted that tumor cell PD-1 levels were positively correlated with poorer prognosis, further underlining the clinical value of tumor PD-1.

Nevertheless, tumor cell-intrinsic PD-1 has been found to depress malignancies in several lung cancer studies (**Figure 2B**). Shisuo Du et al. described a NSCLC patient with hyperprogression after palliative radiotherapy and pembrolizumab treatment, and tumor biopsy found PD-1 positive NSCLC cells (97). Increased viability of PD-1^+^-NSCLC cells M109 following PD-1 blockade was measured *in vitro*. Anti-PD-1 could significantly promote M109 growth in NSG. Yunlong Zhao et al. reported that PD-1 and PD-L1 were co-expressed in NSCLC subpopulations (98). They found that co-expressed PD-1 bound to PD-L1 in cis and inhibited PD-L1 to bind T-cell-PD-1 in trans to repress canonical PD-1/PD-L1 signaling. Selective blockade of tumor-intrinsic PD-1 could release tumor PD-1 to inhibit T-cell function. Xiaodong Wang et al. identified four lung cancer cell lines and 2/7 NSCLC patients expressing PD-1 protein (100). They demonstrated that in an immune-free condition, knockdown/overexpression of PD-1 in tumor cells altered AKT and ERK1/2 phosphorylation dependent on PD-L1, while Nivolumab and Pembrolizumab administration activated AKT and ERK1/2 signaling to promote the growth of PD-1^+^ lung cancer cells and even colon cancer cells. This suggests that the anti-tumor function of PD-1 may not be confined to NSCLC. Zhijie Cao et al. unexpectedly identified that the acetylated p53 preferentially recruited the transcriptional co-activator p300/CBP/TIP60 to the promoter region of PD-1 and elevated the accessibility of PD-1 transcription by upregulating the local histones H3K18/27 and H4K16 acetylation (101). PD-1 in cancer cells inhibited NSCLC (H1299) tumor growth, whereas interference with PD-1 transcriptional activation significantly attenuated the p53-dependent tumor suppression, confirming the materiality of the p53-PD-1 axis. These findings imply that anti-PD-1 for PD-1^+^ NSCLC patients may result in tumor hyperprogression. However, a recent study has suggested that PD-1 expression in lung cancer cell lines (A549, H1975, H1299 and HCC827) can enhance their proliferation and clone formation (102). Therefore, the multifaceted effects of PD-1 on NSCLC still need further research and debate.

## Myeloid Cell-Intrinsic PD-1

Soon after PD-1 was identified, Tasuku Honjo’s team noted that PD-1 was also expressed on myeloid cells (103). Studies have confirmed that PD-1 is expressed on monocytes (104–106), macrophages (79, 107–113), DCs (114–119) and myeloid-derived suppressor cells (MDSCs) (120). Myeloid-PD-1 was markedly upregulated during infections, tumor progression, organ injury and compound induction. Researchers have found that toll-like receptor (TLR) agonists, NOD-like receptor agonists, cytokines, and growth factors all augmented myeloid-PD-1 expression dependent on NF-κB and STAT3, etc. Alexander P. R. Bally and colleagues revealed an NF-κB binding site located in conserved region C upstream of PDCD1 was required for NF-κB-dependent macrophages PD-1 induction (109). Sorim Nam et al. also noticed that PD-1 of MDSCs was regulated by the NK-κB signaling (120). Purushottam Lamichhane et al. found that IL-10 mediated increase in PD-1 of DCs was STAT3 dependent (119). Besides, histone modifications in the PD-1 promoter region are also involved in myeloid-PD-1 induction (109, 112). However, controversy remains in these studies. For example, Sheng Yao et al. reported that PD-1 of spleen DCs was inhibited by TLR9 agonists but not affected by IL-6 and TNF-α (114), but Elias A Said et al. found that TLR9 agonists, IL-6 and TNF-α all promoted PD-1 upregulation in monocytes (104). The differential responses of inducers may be due to cell types and microenvironment, emphasizing that much work remains to be done to investigate the regulatory mechanisms of myeloid-PD-1.

PD-1 engagement affects the differentiation, maturation, survival, metabolism, and effects of myeloid cells (**Figure 2C**). Myeloid-PD-1 altered the balance of differentiation into monocyte and granulocyte cells (106). PD-1 suppressed M1 polarization by reducing STAT1 and NF-κB phosphorylation and promoted M2 polarization by increasing STAT6 phosphorylation (108, 110, 121). Classical phosphorylation of ITIM and ITSM of PD-1 and recruitment of SHP-2 remained upstream of these signals (110). But PD-1 on DCs suppressed antigen presentation *via* MHC I expression inhibition dependent on the NF-κB pathway but independent of SHP-2 (117). In addition to host/tumor cells PD-L1 acting on PD-1^+^ macrophages, T-cell PD-L1 ligation induced M2 differentiation (79). Notably, studies have observed that anti-PD-1 promoted M1 polarization, which may directly function on PD-1^+^ macrophages besides the indirect effect of PD-1^+^ T-cells (122–124). PD-1 ligation on myeloid cells hampered glycolysis and cholesterol metabolism (105, 106). In addition, PD-1-deficient DCs exhibited prolonged longevity dependent on increased MAPK1 and CD40 signaling, as well as maturation-promoting and increased cytokines and co-stimulatory molecules expression, consequently promoting antigen-specific T-cells activity (114–116, 118). Similar phenomena have been observed in monocytes/macrophages (104, 107).

Myeloid-PD-1 expression has been shown in ovarian cancer (115), melanoma (125), gastric cancer (121), NSCLC (98), pleural mesothelioma (112), etc, and generally increased with tumor progression. Tumor-infiltrating PD-1^+^ myeloid cells exhibited immunosuppressive phenotypes with upregulated of PD-L1 and IL-10 and could directly inhibit anti-tumor T-cells infiltration or effects *via* the PD-1/PD-L1 axis (115, 119). New mechanisms of myeloid-PD-1 involvement in tumor immunity have been unearthed in recent years. Sydney R. Gordon et al. found that tumor-associated macrophages (TAMs) PD-1 expression impeded phagocytic potency against tumor cells, and blockade of PD-1 increased phagocytosis and reduced oncogenesis dependent on macrophages (111). Yunlong Zhao et al. reported that co-expressed PD-1 bound to PD-L1 in cis on APCs to hinder PD-L1 acts on T-cell-intrinsic PD-1 in trans (98). The work of Laura Strauss et al. focused on how myeloid-PD-1 affected myeloid cell differentiation, metabolism and effects, particularly during cancer-driven emergency myelopoiesis (79). They discovered a significant reduction in granulocyte/macrophage progenitors (GMP) in PD-1-deficient mice, and myeloid cells of tumor-bearing mice were skewed toward the LY6C^+^ monocytic lineage, which was determined by myeloid-PD-1 deletion. PD-1 deficiency or blockade suppressed monocytic immunosuppressive functions. Myeloid-PD-1-knockout was superior to systemic PD-1-knockdout and T-cell PD-1 conditional knockout for tumor inhibition, even in MC38 tumors where T-cells PD-1 knockout functioned slightly but myeloid-PD-1 deletion completely inhibited MC38 growth. Notably, anti-PD-1 antibodies were still effective in mice lacking T-cells.

Thus, the role of myeloid-PD-1 in anti-PD-1 therapy is gaining attention, and several combination strategies have been proposed. Purushottam Lamichhane et al. found that DCs responded to PD-1 blockade by increasing IL-10 production (119). The combination of PD-1 and IL-10 blockade significantly reduced tumor burden. Hirotake Tsukamoto et al. found that blocking PD-1/PD-L1 prompted PD-1^+^ TAMs to produce IL-6. Depletion of macrophages in melanoma-bearing mice reduced the levels of IL-6 during PD-1/PD-L1 blockade, suggesting that IL-6-neutralizing antibodies are potential candidates for combination with anti-PD-1 antibodies (125). In addition, inhibition of EZH2 methyltransferase was found to promote PD-1 expression on macrophages, and the combination of EZH2 inhibitors and anti-PD-1 antibodies could achieve better anti-tumor efficacy (112).

## Other Noncanonical PD-1/PD-L1 Expression

In addition to the above discussion, unacquainted PD-1/PD-L1 expression in other cell types also requires attention. Taking NK cells as an example, although less studied, available reports have supported that NK cells can express PD-1/PD-L1. PD-L1 engagement can inhibit PD-1^+^ NK cell-mediated antitumor responses (126, 127). Increased NK cell PD-1 expression is associated with tumor progression and poor prognosis in patients (128, 129). Anti-PD-1 treatment can promote NK cell activation, intratumoral recruitment, and anti-tumor cytotoxicity (130–132). Studies on PD-L1 in NK cells are much rarer. Existing results suggest that the TME can upregulate PD-L1 in NK cells (129, 133). PD-L1 inhibitors can not only block the inhibitory signal of PD-1, surprisingly, also directly activate PD-L1^+^ NK cells (133).

Furthermore, the expression of PD-1/PD-L1 in other non-immune cells may also affect the efficacy or safety of anti-PD therapy. For example, PD-1 was found to be expressed by primary sensory neurons in the dorsal root ganglion and to affect their signaling, and administration of anti-PD-1 antibodies to mice or non-human primates led to altered opioid-induced antinociception (134).

## Discussion of Noncanonical PD-1/PD-L1 Axis Associated Therapy Strategies

Shifting the focus of anti-PD therapies from the classical PD-1/PD-L1 axis to noncanonical axis may provide opportunities to broaden the benefits of PD-1/PD-L1 blockade through rational drug design and combination based on the regulatory role of noncanonical PD-1/PD-L1 axis in tumorigenesis.

The involvement of T-cell intrinsic PD-L1 in immunosuppression is increasingly recognized as an additional mechanism for anti-PD-L1 efficacy. Considering several studies emphasized that many anti-PD-L1 antibodies could trigger apoptosis of PD-L1^+^ T-cells (72, 77), excluding such antibodies *via* T-cell apoptosis assays and using of Fc with weak effects are spurred. Besides, potential cis-interactions of PD-1/PD-L1 and CD80/PD-L1 on T-cells need to be investigated. They have been shown to contend with PD-1/PD-L1 and CD80/CTLA-4 trans-interactions (54, 98), so that anti-PD-1/PD-L1 antibodies alone lead to the release of inhibitory signals after breaking cis-interactions. Coadministration of anti-PD-1, anti-PD-L1 and anti-CTLA-4 antibodies or treatment of anti-PD-1/PD-L1/CTLA-4 trispecific antibodies may be candidate approaches to completely unleash innate and adaptive immunity to eradicate tumors, which also fits well with cancers with PD-1^+^ tumor or myeloid cells.

Intracellular signals of tumor-intrinsic PD-1 as accomplices of malignancies are candidate strategies for combination with anti-PD-1 antibodies, such as Hippo and mTOR pathways. Notably, PD-1 of tumor cells has been found to depress NSCLC tumor growth, and anti-PD-1 treatment may even lead to tumor hyperprogression. In general, anti-PD therapies result in significant activation of T-cells in patients to eliminate tumors. However, in immunocompromised patients with low initial activated T-cells, anti-PD antibodies administration could not normalize the intratumoral T-cells function, but may raise the pro-tumor signaling, thus leading to tumor hyperprogression (21). Therefore, for NSCLC or cancers with hyperprogression caused by immunotherapies, caution is needed for anti-PD-1/PD-L1 treatment. Combination with AKT and ERK1/2 inhibitors is an approach to contain the tumor-promoting signaling activated by anti-PD-1 antibodies (100), and combination with innate immune agonists can boost the antitumor responses of patients. Combining them may be a beneficial strategy for patients with hyperprogression.

For myeloid cells, increased secretion of IL-6/IL-10 induced by anti-PD-1 antibodies is also a potential target for combination to further repress the alternative inhibitory molecules (119, 125). In addition, the noncanonical expression of other checkpoint molecules such as lymphocyte activation gene-3 (LAG-3) (135, 136) and T cell immunoglobulin domain and mucin domain-3 (TIM-3) (137) on myeloid cells also needs attention and investigation, and combination with these checkpoint inhibitors holds promise for overcoming antitumor resistance.

## Conclusion and Perspectives

In summary, the noncanonical PD-1/PD-L1, represented by T-cell-intrinsic PD-L1, tumor cell-intrinsic PD-1, and myeloid PD-1, exhibits unique protein interactions, signaling and cell crosstalk to regulate cell growth, differentiation, metabolism and effects dependently on immunity or not. But noncanonical signaling contributes to both anti-PD efficacy and resistance, and further studies are needed to resolve, balance, or even exploit these controversies for clinical applications of the noncanonical PD-1/PD-L1 axis. Integrating the classical and non-classical PD-1/PD-L1 axes and revisiting the role of the holistic PD-1/PD-L1 axis on tumor progression in specific cancer types and stages, improved therapeutic efficacy and safety of anti-PD therapies will be achieved through rational drug design and combination.

## Author Contributions

YL and LG conceived the topic. YL and XY drafted the manuscript and prepared the figures. Others reviewed the manuscript. All authors read and approved the final manuscript. All authors contributed to the article and approved the submitted version.

## Funding

This work was supported by Foundation of Shanghai Science and Technology Committee (No. 22S11902100), Zhongshan Municipal Bureau of Science and Technology (No. 2020SYF08) and the Strategic Priority Research Program of the Chinese Academy of Sciences (No. XDA 12050305).

## Conflict of Interest

The authors declare that the research was conducted in the absence of any commercial or financial relationships that could be construed as a potential conflict of interest.

## Publisher’s Note

All claims expressed in this article are solely those of the authors and do not necessarily represent those of their affiliated organizations, or those of the publisher, the editors and the reviewers. Any product that may be evaluated in this article, or claim that may be made by its manufacturer, is not guaranteed or endorsed by the publisher.
